# Identification of early risk factors for anti-citrullinated-protein-antibody positive rheumatoid arthritis—a prospective cohort study

**DOI:** 10.1093/rheumatology/keae146

**Published:** 2024-03-08

**Authors:** Alexandra Cîrciumaru, Yogan Kisten, Monika Hansson, Linda Mathsson-Alm, Vijay Joshua, Heidi Wähämaa, Malena Loberg Haarhaus, Joakim Lindqvist, Leonid Padyukov, Sergiu-Bogdan Catrina, Guozhong Fei, Nancy Vivar, Hamed Rezaei, Erik af Klint, Aleksandra Antovic, Bence Réthi, Anca I Catrina, Aase Hensvold

**Affiliations:** Division of Rheumatology, Department of Medicine Solna, Karolinska Institutet, Stockholm, Sweden; Center for Rheumatology, Academic Specialist Center, Stockholm Health Services, Region Stockholm; Division of Rheumatology, Department of Medicine Solna, Karolinska Institutet, Stockholm, Sweden; Division of Rheumatology, Department of Medicine Solna, Karolinska Institutet, Stockholm, Sweden; Thermo Fisher Scientific, Uppsala, Sweden; Thermo Fisher Scientific, Uppsala, Sweden; Division of Rheumatology, Department of Medicine Solna, Karolinska Institutet, Stockholm, Sweden; Division of Rheumatology, Department of Medicine Solna, Karolinska Institutet, Stockholm, Sweden; Division of Rheumatology, Department of Medicine Solna, Karolinska Institutet, Stockholm, Sweden; Department of Rheumatology, Karolinska University Hospital; Division of Rheumatology, Department of Medicine Solna, Karolinska Institutet, Stockholm, Sweden; Department of Rheumatology, Karolinska University Hospital; Division of Rheumatology, Department of Medicine Solna, Karolinska Institutet, Stockholm, Sweden; Department of Molecular Medicine and Surgery, Karolinska Institutet, Stockholm, Sweden; Center for Diabetes, Academic Specialist Centrum, Stockholm, Sweden; Center for Rheumatology, Academic Specialist Center, Stockholm Health Services, Region Stockholm; Swedish Medical Products Agency, Uppsala, Sweden; Division of Rheumatology, Department of Medicine Solna, Karolinska Institutet, Stockholm, Sweden; Department of Rheumatology, Karolinska University Hospital; Division of Rheumatology, Department of Medicine Solna, Karolinska Institutet, Stockholm, Sweden; Department of Rheumatology, Karolinska University Hospital; Division of Rheumatology, Department of Medicine Solna, Karolinska Institutet, Stockholm, Sweden; Department of Rheumatology, Karolinska University Hospital; Division of Rheumatology, Department of Medicine Solna, Karolinska Institutet, Stockholm, Sweden; Center for Rheumatology, Academic Specialist Center, Stockholm Health Services, Region Stockholm; Department of Rheumatology, Karolinska University Hospital; Division of Rheumatology, Department of Medicine Solna, Karolinska Institutet, Stockholm, Sweden; Division of Rheumatology, Department of Medicine Solna, Karolinska Institutet, Stockholm, Sweden; Center for Rheumatology, Academic Specialist Center, Stockholm Health Services, Region Stockholm; Department of Rheumatology, Karolinska University Hospital; Division of Rheumatology, Department of Medicine Solna, Karolinska Institutet, Stockholm, Sweden; Center for Rheumatology, Academic Specialist Center, Stockholm Health Services, Region Stockholm

**Keywords:** rheumatoid arthritis, risk phase, biomarkers, anti-citrullinated protein antibody reactivities

## Abstract

**Objective:**

Individuals positive for anti-cyclic-peptide-antibodies (anti-CCP) and musculoskeletal complaints (MSK-C) are at risk for developing rheumatoid arthritis (RA). In this study we aimed to investigate factors involved in arthritis progression.

**Methods:**

Anti-CCP2-positive individuals with MSK-C referred to a rheumatologist were recruited. Individuals lacked arthritis at clinical and ultrasound examination and were followed for ≥3 years or until clinical arthritis diagnosis. Blood samples from inclusion were analysed for nine ACPA reactivities (citrullinated α-1-enolase, fibrinogen, filaggrin, histone, vimentin and tenascin peptides); 92 inflammation-associated proteins; and HLA-shared epitope alleles. Cox regression was applied to the data to identify independent predictors in a model.

**Results:**

Two hundred and sixty-seven individuals were included with median follow-up of 49 months (interquartile range [IQR]: 22–60); 101 (38%) developed arthritis after a median of 14 months (IQR: 6–27). The analysis identified that presence of at least one ACPA reactivity (hazard ratio [HR] 8.0; 95% CI: 2.9, 22), ultrasound-detected tenosynovitis (HR 3.4; 95% CI: 2.0, 6.0), IL-6 levels (HR 1.5; 95% CI: 1.2, 1.8) and IL-15 receptor α (IL-15Rα) levels (HR 0.6; 95% CI: 0.4, 0.9) are significant independent predictors for arthritis progression in a prediction model (Harrell’s *C* 0.76 [s.e. 0.02], AUC 0.82 [95% CI: 0.76, 0.89], cross-validated AUC 0.70 [95% CI: 0.56, 0.85]).

**Conclusion:**

We propose a high RA risk phase characterized by presence of ACPA reactivity, tenosynovitis, IL-6 and IL-15Rα and suggest that these factors need to be further investigated for their biological effects and clinical values, to identify individuals at particular low risk and high risk for arthritis progression.

Rheumatology key message

- ACPA reactivities, tenosynovitis and inflammation proteins (IL-6 and IL-15Rα) are important risk factors for progression to arthritis.

## Introduction

Seropositive rheumatoid arthritis (RA) is caused by a complex interaction between genes and environmental factors [1, 2]. Environmental risk and protective factors are of great importance, and a majority (9 out of 10) of patients lack any close relative with RA [3]. At diagnosis onset, a typical RA patient presents with joint inflammation assessed clinically, by ultrasound or MRI (EULAR/ACR RA classification criteria 2010 [4]), and autoantibodies against different types of post-translationally modified proteins, such as ACPA and rheumatoid factor (RF). Results from retrospective studies in Pre-RA cohorts have shown that both anti-cyclic-citrulline peptide antibodies (anti-CCP), other ACPA reactivities and increase of some inflammatory markers can precede RA diagnosis and/or symptom onset with a median time of 5–6 years (maximum 13–25 years) [5–8]. These factors have, however, not been investigated in detail in prospective Risk-RA cohorts. Specific ACPA reactivities have been suggested to have early disease mechanistic roles by increasing osteoclast activation and subsequently bone destruction and pain-like behaviour in the absence of clinical inflammation, as detected in experimental mouse models [9–13]. RF has also been suggested to have disease-inducing effects with potential synergies with ACPA [14].

Prospective Risk-RA cohort studies have confirmed the existence of a risk phase with systemic autoimmunity and with or without musculoskeletal complaints. Anti-CCP-positive individuals, who lack signs of microscopic synovial inflammation [15, 16] or clinically manifest joint inflammation, are at increased risk for RA, but risk estimates vary between different studies. A summary of results from previous cohort studies with different inclusion criteria is provided in Supplementary Table S1, available at *Rheumatology* online. In these studies, the risk for arthritis development varied between 6% and 61% (with median 33%).

In order to contribute to the understanding of the Risk-RA phase, we investigated the Risk-RA cohort at Karolinska Institutet. Our more specific aim was to increase understanding about biological factors that affect risk for, or protection from, arthritis; this can eventually be used to identify individuals having very high or low risk for development of arthritis. For this purpose, we studied multiple immunological and inflammatory factors by measuring ACPA reactivities and inflammation-associated proteins, as well as clinical factors, e.g. ultrasound joint investigations.

## Methods

### Karolinska Risk-RA prospective research programme and cohort

The Karolinska Risk-RA is a prospective research programme that started in 2014, recruiting individuals from all three rheumatology clinics in Region Stockholm, Sweden: Karolinska University Hospital, Academic Specialist Center and Danderyd Hospital.

Individuals are eligible for participation if they: (i) have musculoskeletal complaints referred with suspicion of rheumatic disease from a primary care specialist (93%) or other specialist (7%) and after a rheumatologist’s assessment; (ii) are screened for no prior/current rheumatic diseases; (iii) are positive for anti-CCP2-test; and (iv) lack signs of arthritis assessed by ultrasound.

Participation in this prospective research programme includes at least three annual follow-up visits, until clinical arthritis develops, or at least 3 years have passed. Individuals are offered direct telephone contact if symptoms become worse and on-demand visits if needed (for further details see Supplementary Data S1, available at *Rheumatology* online). Anti-CCP2 IgG and RF IgM were analysed in a routine clinical laboratory and were defined as positive if above upper limit of normal according to the clinical laboratory. Arthritis diagnosis is defined clinically by a trained rheumatologist (joint swelling at physical examination), and in the vast majority the joint swelling is verified by ultrasound. This study includes 267 individuals enrolled between May 2014 and April 2019, with data collected up to May 2022 (Supplementary Fig. S1, available at *Rheumatology* online). Median follow-up of the individuals was 49 months (interquartile range [IQR]: 22–60). The study is conducted in accordance with the Declaration of Helsinki, with approval from the Swedish Ethical Review Authority. All study participants provided written informed consent before study enrolment.

### Musculoskeletal ultrasound assessment

Hands and feet along with additional symptomatic joints were evaluated by musculoskeletal ultrasound (MSUS) examination using EULAR-OMERACT [17] definitions and scoring of synovitis. Individuals having at least moderate synovial hypertrophy (grey scale (GS) ≥2 and/or colour Doppler (CD) ≥1) were excluded. Baseline data from individuals with GS ≤ 1 and CD = 0, on eventual presence of minimal synovial hypertrophy (GS = 1 without CD), tenosynovitis and bursitis, were collected and analysed. A detailed MSUS protocol is available in Supplementary Data S1, available at *Rheumatology* online.

### Analysis of autoantibodies, genetic risk alleles and inflammation-associated proteins

Sera samples from baseline were utilized. Nine ACPA reactivities targeting citrullinated peptides from α-1-enolase, fibrinogen, filaggrin, histone, vimentin and tenascin (Supplementary Table S2, available at *Rheumatology* online), along with their corresponding native arginine control peptides, were analysed using an custom-made multiplex solid phase microarray platform (Thermo Fisher Scientific, ImmunoDiagnostics Division, Uppsala, Sweden), as described previously [18]. HLA-DRB1 shared epitope (SE) genotyping was performed using a DR low resolution kit, and when needed to discriminate risk alleles high resolution kits were used (Thermo Fisher Scientific, Waltham, MA, USA). HLA DRB1 *01:01, *01:02, 04:01, *04:04, 04:05 and *10:01 were defined as HLA-SE (risk) alleles [19]. A panel of 92 serum inflammation-associated proteins was analysed using a multiplex immunoassay with proximity extension technology (O-link Bioscience, Uppsala, Sweden) in the Risk-RA cohort and in an additional cohort of healthy controls matched by age, sex and sample handling (*n* = 140, mean age 53 years; 76% females). Serum protein values above the lower detection limit (*n* = 78 proteins) were selected to be used for the analysis and are presented as normalized protein expression (NPX), an arbitrary unit on a log2 scale.

### Statistical analysis

Baseline data were normalized when appropriate. RF and anti-CCP2 levels were normalized to the respective cut-off values used in the clinical laboratory and defined as high if the titre was >3× upper limit of normal [4]. Kaplan–Meier analysis and log-rank test were performed to analyse the survival time (arthritis free). Significant baseline factors among clinical parameters, autoantibodies, ultrasound parameters and inflammation-associated proteins that associated with progression to arthritis were identified in univariate Cox regression analysis. Collinearity between significant baseline factors was investigated by analysis of correlations, and candidate variables for the prediction models were selected. Significance-based forward and backward selection of the candidate variables (*n* = 10) was used to determine significant independent predictors for arthritis. The final regression model is presented with Harrell’s *C*, area under the curve (AUC), and internally cross validated using the ‘leave-one-out cross-validation method’ [20]. Interaction between final predictors is also explored. Complete case analysis was performed, and eventual effect of incomplete data was tested by incomplete case analysis. Statistical analyses were performed with SAS v.9.3 (SAS Institute, Cary, NC, USA) and R Studio software, and the level of significance was set as 0.05. GraphPad Prism v 9.1.0 software (GraphPad Software, Boston, MA, USA) was used for graphs and images. We used the TRIPOD checklist when writing our report [21]. Further details of the statistical analysis are available in Supplementary Data S1, available at *Rheumatology* online.

## Results

### Baseline characteristics of the study cohort

A total of 267 individuals were included in this study (Supplementary Fig. S1, available at *Rheumatology* online, Table 1), with a mean age of 48 years (s.d. 14) and 210 (79%) female. Median symptom duration prior to inclusion in the study was 21 months (IQR: 10–51); 148/256 (58%) of participants had ever smoked tobacco and 37 (25%) were current smokers at the time of inclusion; 68/255 (27%) had a first degree relative (parent, sibling and/or offspring) with a RA diagnosis. HLA-SE alleles were present in 165/254 (65%) of individuals: 119 (47%) with one allele; 46 (18%) with two alleles.

**Table 1.** Clinical characteristics at baseline of the Karolinska Risk-RA cohort and association with arthritis progression

Variable^a^	Risk-RA cohort (*n* = 267)	Arthritis free (*n* = 166)	Progressors (*n* = 101)	HR (95% CI)	*P*-value
Follow-up duration, median (IQR), months	49 (22–60)	54 (49–68)	14 (6–27)		
Age, mean (s.d.), years	48 (14)	47 (15)	50 (13)	1.0 (1.0, 1.0)	0.12
Female, *n* (%)	210 (79)	128 (77)	82 (81)	1.2 (0.7, 2.0)	0.43
FDR with RA, *n* (%)	68 (27)	40 (25)	28 (29)	1.1 (0.7, 1.7)	0.60
Ever smoker, *n* (%)	148 (58)	87 (54)	61 (64)	1.3 (0.9, 2.0)	0.18
Symptom duration, median (IQR), months	21 (10–51)	25 (11–62)	13 (7–37)	1.0 (1.0, 1.0)	0.06
BMI, median (IQR), kg/m^2^	25 (22–28)	25 (22–28)	25 (23–27)	1.0 (0.9, 1.0)	0.43
ESR mm/h, median (IQR)	11 (5–19)	10 (5–20)	12 (6–18)	1.0 (1.0, 1.0)	0.98
General health, median (IQR), VAS	28 (7–51)	24 (4–54)	30 (10–47)	1.0 (1.0, 1.0)	0.91
Pain, median (IQR), VAS	27 (10–52)	25 (9–52)	31 (10–52)	1.0 (1.0, 1.0)	0.45
Fatigue, median (IQR), VAS	29 (3–60)	26 (4–62)	35 (1–58)	1.0 (1.0, 1.0)	0.81
Number tender 28 joints, median (IQR)	0 (0–2)	0 (0–2)	0 (0–2)	1.0 (0.9, 1.0)	0.76
RF positive, *n* (%)	88 (33)	37 (22)	51 (51)	2.7 (1.7, 3.8)	<0.0001
HLA-SE carriers (≥1 allele), *n* (%)	165 (65)	92 (58)	73 (77)	2.1 (1.3, 3.3)	0.003

a Missing data for FDR (12), ever smoker (11), BMI (45), ESR (17), VAS general health (34), VAS pain (34), VAS fatigue (68), TJC28 (3), HLA-SE (13). FDR: first degree relative (parents, siblings and/or offspring); HLA-SE: human leucocyte antigen shared epitope; IQR: interquartile range; TJC: tender joint count; VAS: visual analogue scale.

All individuals were anti-CCP2 positive at inclusion, with a median titre 9.3 (IQR: 2.6–90) times above cut-off (AU/ml); RF was present in 88 (33%) individuals, with median titre 3.3 (IQR: 1.6–4.9) times above cut-off (IU/ml); 190/252 (75%) of individuals were positive for ≥1 of 9 ACPA reactivities tested. The musculoskeletal complaints among individuals were assessed, resulting in an overall median of 0 (IQR: 0–2) for tender 28-joint counts, and 27 (IQR: 10–52) for visual analogue scale for pain.

### Clinical characteristics at baseline and association with arthritis onset

**Figure 1.**
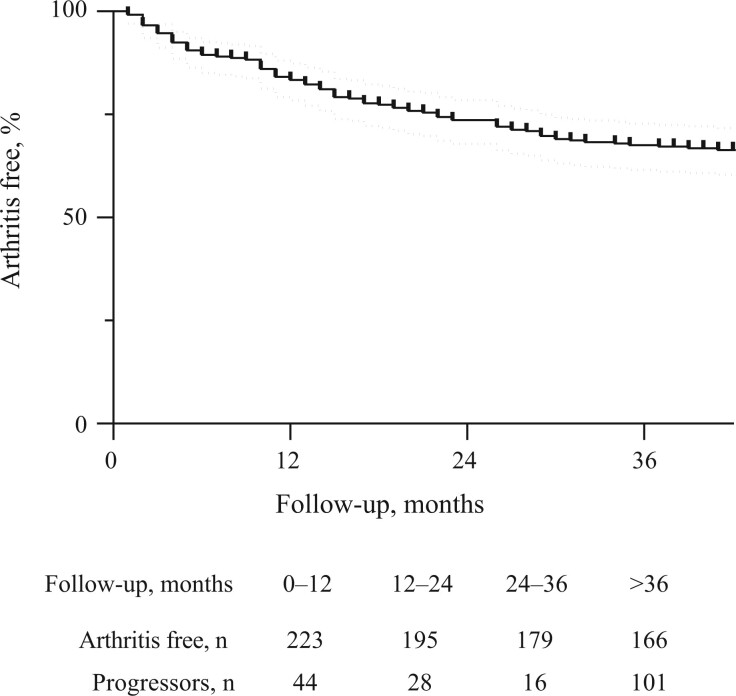
Kaplan–Meier curve showing arthritis free survival time in Risk-RA individuals. Dotted line represents CI 95%

Progression towards arthritis was observed in 101 individuals (38%) after a median of 14 months of follow-up (IQR: 6–27): 44 (16%) within 1 year; 28 (13%) between 1 and 2 years; 16 (8%) between 2 and 3 years; and 13 (7%) after >3 years follow-up (Fig. 1). At arthritis onset 91 (90%) individuals had classifiable RA according to the 2010 criteria and 85 (84%) initiated disease-modifying-anti-rheumatic-drugs.

We compared the clinical characteristics of the individuals who developed arthritis to those who did not (Table 1). RF positivity was found in 51 out of 101 (50%) individuals who developed arthritis, compared with 37 out of 166 (22%) who did not develop arthritis (HR 2.7; 95% CI: 1.7, 3.8; *P* < 0.0001). RF levels were non-significantly increased in individuals developing arthritis (*P* > 0.05; Supplementary Fig. S2, available at *Rheumatology* online). HLA-SE alleles (≥1) were present in 73 out of 95 individuals (77%) who developed arthritis, compared with 92 out of 159 individuals (58%) who did not (HR 2.1; 95% CI: 1.3, 3.3; *P* = 0.003). Ever regular smoker or current regular smoker were non-significantly associated to arthritis progression (respectively HR 1.3; 95% CI: 0.9, 2.0; *P* = 0.18; and HR 1.5; 95% CI: 0.9, 2.5; *P* = 0.13). We further investigated effects of other clinical characteristics, e.g. self-reported symptom assessments, and found no association to arthritis progression.

### Anti-citrullinated protein antibodies at baseline and association with arthritis onset

**Figure 2.**
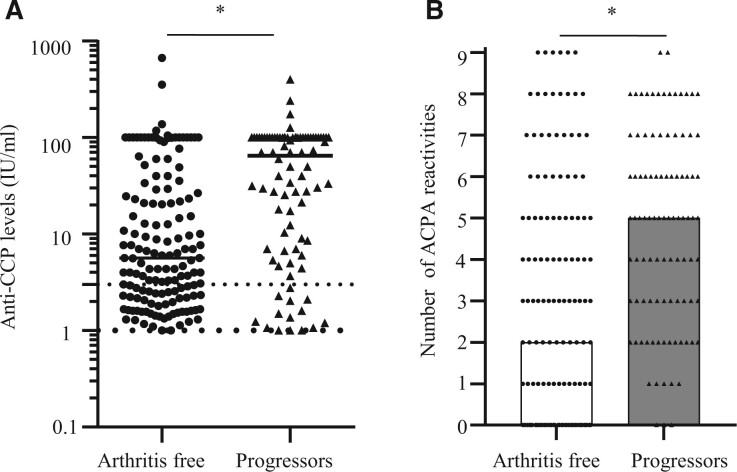
Anti-citrullinated protein antibodies (ACPA) in individuals who progressed towards arthritis, compared with individuals who remained arthritis free. (**A**) Anti-CCP levels as measured by anti-CCP test; lower dotted line represents upper limit of normal value and cut-off for positive test, and the upper dotted line represents high level (≥3× upper limit of normal). *n* = 267, *P = 0.0002, univariate Cox regression. (**B**) Number of ACPA reactivities (range 0–9). *n* = 252, *P < 0.001, univariate Cox regression

Anti-CCP levels were increased in individuals developing arthritis (*P* = 0.0002, Fig. 2A). Associations with arthritis progression were also found for anti-CCP high (≥3× upper limit of normal) levels (HR 2.1; 95% CI: 1.2, 3.54.0, *P* = 0.006).

Progression towards arthritis was associated with increased number of ACPA reactivities (median 7, IQR: 4–9; *P* < 0.0001, Fig. 2B). CEP-1, Cit-Fibβ_36–52,_ Cit-Fil_307–324_, Cit-His3_21–44_, Cit-TNC1, Cit-TNC5, Cit-Vim_2–17_ and Cit-Vim_60–75_ antibodies were significantly more prevalent in progressors, as compared with non-progressors to arthritis (Supplementary Table S3, available at *Rheumatology* online). The strongest association with progression was identified as the presence of ≥1 of the analysed ACPA reactivities (HR 10; 95% CI: 3.8, 28; *P* < 0.0001). The combination of ≥1 ACPA reactivity and RF positivity (HR 2.5; 95% CI: 1.7, 3.8; *P* < 0.0001) did not perform better as a predictive factor than ≥1 ACPA reactivity alone.

### Minimal ultrasound changes at baseline and association with arthritis onset

Ultrasonographic changes of presence of tenosynovitis, minimal synovial hypertrophy (GS ≤ 1, CD = 0), presence of bursitis or any of the above at baseline was found in 19 (7%), 20 (8%), 6 (2%) and 43 (17%) individuals, respectively. When comparing ultrasound data from the individuals who developed arthritis with those who did not, we found a significant difference in the presence of tenosynovitis. Tenosynovitis was observed in 16 (16%) individuals who progressed towards arthritis, compared with three (2%) who remained arthritis free (*P* < 0.0001; Supplementary Table S4, available at *Rheumatology* online). Sixteen out of 19 individuals with tenosynovitis progressed to arthritis, compared with 79 out of 234 individuals without tenosynovitis who progressed to arthritis (84% *vs* 34%, respectively).

### Inflammation-associated proteins at baseline and association with arthritis onset

There were similar levels for 68 out of 78 (87%) of the inflammation-associated proteins analysed between those developing arthritis and individuals not developing arthritis. There were also similar levels of 35 (45%) proteins and significantly different levels of 43 (55%) proteins in Risk-RA individuals compared with healthy controls. Significantly different levels of 10 proteins (IL-6, TNF-R superfamily member 9, IL-17C, IL-10, CXCL9, IL-15 receptor α [IL-15Rα], CXCL6, leukaemia inhibitory factor receptor [LIF-R], delta and Notch-like epidermal growth factor-related receptor [DNER] and hepatocyte growth factor [HGF]) between those developing arthritis and individuals not developing arthritis (Supplementary Table S5, Supplementary Fig. S3, available at *Rheumatology* online).

### Significant independent predictors in a model for arthritis progression

We investigated all significant factors for correlation and performed multivariate Cox regression with the 10 selected candidate significant factors: anti-CCP levels, combined RF status and anti-CCP levels, number of HLA-SE alleles, ≥1 ACPA reactivity, ultrasound-detected tenosynovitis, IL-6, IL-17C, DNER, IL-15Rα and CXCL6 (Supplementary Tables S6 and S7, available at *Rheumatology* online). We identified independent predictors in a prediction model (Harrell’s *C* 0.76 [s.e. 0.02], AUC 0.82 [95% CI: 0.76, 0.89]; Supplementary Tables S8 and S10, available at *Rheumatology* online). We tested for interaction, but this did not add significant value to the model (data not shown). We also performed sensitivity analysis including individuals with incomplete data (*n* = 260), with minor effect (Supplementary Table S9, available at *Rheumatology* online); and internal cross-validation of the identified prediction model (AUC 0.70; 95% CI: 0.56, 0.85; Supplementary Table S10, available at *Rheumatology* online). In addition, we tested the model after adjustment by age and sex (data not shown) and did sensitivity analysis including arthritis progressors with classifiable RA (Supplementary Table S11, available at *Rheumatology* online) with minor effect.

**Figure 3.**
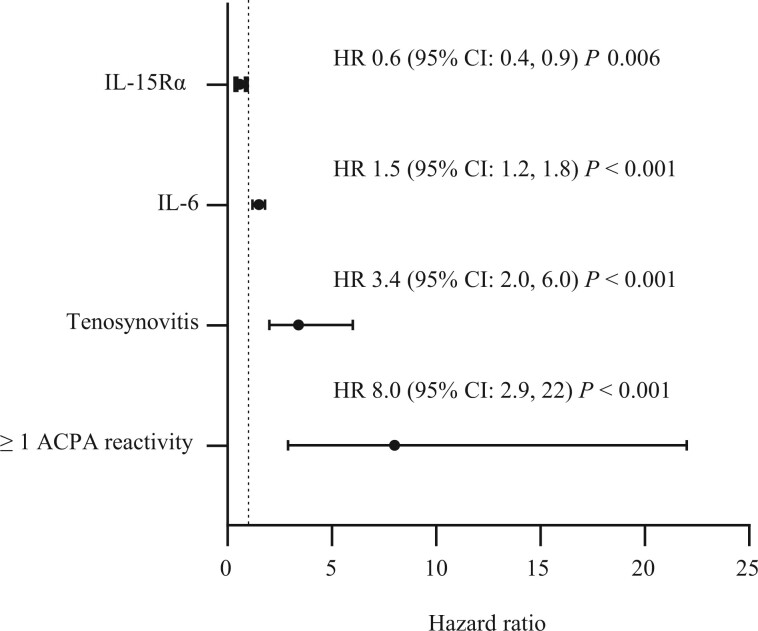
Prediction model for arthritis progression. After multivariate Cox regression modelling ACPA reactivity (i.e. positivity for at least one of the nine tested ACPA reactivities targeting Cit-Fil, Cit-Fibβ, Cit-Vim, CEP-1, Cit-TNC1, Cit-TNC5, Cit-His3 peptides), ultrasound-detected tenosynovitis, IL-6 and IL-15Rα (both measured by OLINK inflammatory panel) were found to be significant independent predictors. Two hundred and thirty-two individuals with complete data were used in the model

**Figure 4.**
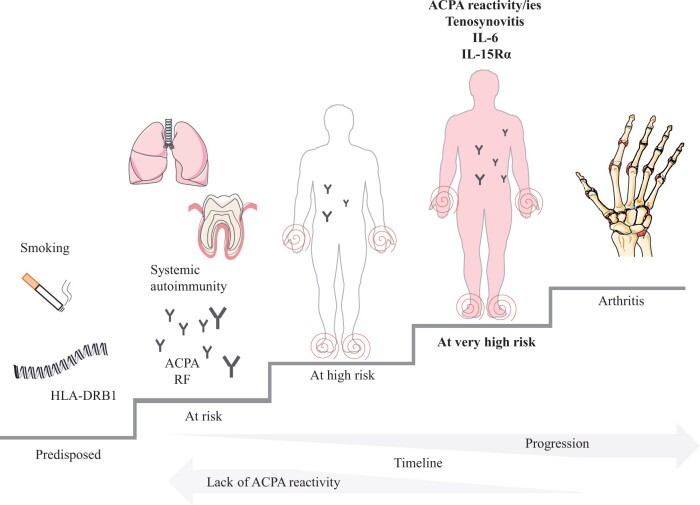
A model for progression towards arthritis. Individuals exposed to smoking and certain risk gene alleles (HLA-DRB1) will be predisposed to immune activation at mucosal sites such as lung and gingiva, and the development of systemic autoimmunity with ACPA and RF. Individuals further developing musculoskeletal complaints are at high risk for arthritis, but many of these individuals do not progress. We propose a revised model where certain ACPA reactivities, levels of IL-6 and IL-15Rα and ultrasound-detected tenosynovitis are factors in a stage of very high risk for arthritis. This figure was in part generated using images adapted from Servier Medical Art by Servier, licensed under a Creative Common Attribution 3.0 unsupported licence

Presence of ≥1 ACPA reactivity, ultrasound-detected tenosynovitis, IL-6 levels and IL-15Rα levels were identified as significant independent predictors for arthritis progression (Figs 3–4) and after utilizing Bonferroni adjustment, these predictors remained significant with the exception of IL-15Rα, which was borderline significant. The diagnostic accuracy for the binary factors identified was explored and compared with testing for RF, see Table 2.

**Table 2.** Diagnostic accuracy of testing any ACPA reactivity, tenosynovitis detected by ultrasound and RF in anti-CCP positive individuals with musculoskeletal symptoms

Test	Result	Progressors	Arthritis free	Sensitivity (95% CI)	Specificity (95% CI)	PPV (95% CI)	NPV (95% CI)
Any ACPA reactivity	Positive	96	94	96% (92, 99.8)	38% (30, 46)	51% (43, 58)	94% (87, 99.7)
	Negative	4	58				
Tenosynovitis	Positive	16	3	17% (9, 24)	99% (97, 100)	89% (74, 100)	64% (57, 70)
	Negative	80	140				
RF positivity	Positive	51	37	51% (41, 61)	76% (69, 82)	58% (48, 68)	70% (63, 77)
	Negative	49	115				

Any positive ACPA reactivity was defined as positive for ≥1 out of nine tested ACPA reactivities. Any negative ACPA reactivity was defined as negative for all nine tested ACPA reactivities. Analysed individuals testing any ACPA reactivity, *n* = 252; tenosynovitis detected by ultrasound, *n* = 239; RF, *n* = 252. NPV: negative predictive value; PPV: positive predictive value.

## Discussion

The aim of this study was to identify factors involved in arthritis progression in anti-CCP-positive individuals referred from primary care due to musculoskeletal complaints (MSK-C), where no signs of joint inflammation could be observed at clinical and ultrasound examination. The study was performed within the Karolinska Risk-RA cohort and comprised baseline data from 267 individuals, who were observed for arthritis development for at least 3 years or until arthritis (joint swelling at physical examination) was diagnosed, which occurred in 101 individuals (38%) at a time point were 91 (90%) had classifiable RA according to the 2010 criteria.

Major findings from the analysis were an increased presence of ACPA reactivities directed towards certain protein-derived citrullinated peptides in the group that progressed to arthritis (96%), as compared with the one that remained arthritis-free (62%); and that the presence of at least one of the tested ACPA reactivities is a strong predictor of arthritis development. Additionally, a proteomics screen of inflammation-associated proteins showed several differences between Risk-RA and healthy controls, and some differences between the arthritis progressors and non-progressors, with notably slightly higher serum IL-6 levels and lower IL-15Rα levels in the group of progressors. Finally, individuals who had ultrasound-detected tenosynovitis at inclusion were almost destined (89%) to develop arthritis. In summary we propose a high-Risk-RA phase characterized by presence of these factors (Fig. 4).

The exposure to environmental and genetic factors in the cohort and the frequency of arthritis are similar to what has been reported in some earlier studies, even with our strict inclusion criteria that excluded moderate and severe synovitis detected by ultrasound and allowing only minimal ultrasound changes at baseline. It is also of interest that presence of SE alleles was significantly associated with arthritis progression, whereas exposure to cigarette smoking was not, although smoking showed a numerical trend for association with disease progression. These observations are in line with the hypothesis suggested in earlier reports from us and others that HLA-SE alleles may act both in the early phase associated with triggering of antibody production and in the presently investigated phase of arthritis development, whereas the relative role of smoking may be greater in the early phase of immune activation [22]. Notably, only a minority of the individuals at risk were RF positive at baseline, with RF being a significant risk factor for progression towards arthritis. This observation agrees with some, but not all, previous reports that anti-citrulline reactivity may develop prior to rheumatoid factors in most patients, thus suggesting that ACPA and immune complexes involving ACPA may be involved in subsequent later triggering of RF production and development of chronic inflammation.

Studies of longitudinal samples from Pre-RA individuals and healthy controls suggest that epitope spreading and increasing number and levels of ACPA reactivity is characteristic for an individual developing ACPA positive RA. An early study by Stadt *et al*. suggested that multiple ACPA reactivities (≥2 *vs* 0–1) was a significant risk factor (odds ratio 2.1; 95% CI: 1.0, 4.4; *P* = 0.04) with a strong trend of prediction for arthritis development (HR: 1.7; 95% CI: 0.93, 3.16, *P* = 0.08) in the Risk-RA phase [23]; and we can in this study report a significant predictive value of presence of ACPA reactivity (≥1 *vs* 0) for arthritis (HR 8.0; 95% CI: 2.9, 22; *P* < 0.001). Like previous Pre-RA studies [5, 24] we could identify particular ACPA reactivities targeting, for example, citrullinated vimentin and enolase with association to arthritis progression, but in comparison presence of at least one ACPA reactivity was in our study a stronger predictive factor.

Results from other studies suggest that there are additional factors, such as anti-carbamylated protein antibodies (significant in univariate but not multivariate analysis) [25, 26] and anti-CCP3 levels [27] that associate with arthritis progression. The lack of data on these factors is a limitation in our study, as is the absence of data on other heterogeneities in the ACPA repertoire, e.g. Fab glycosylation [28, 29] and IgA isotype [30, 31]. However, suggested associated hazard ratios for these factors are relatively low, compared with the effect of presence of ACPA directed towards certain protein-derived citrullinated peptides.

Inflammatory proteins have previously been investigated in Pre-RA cohorts, but no systematic screening of inflammation-associated proteins (*n* = 92) has been performed in a Risk-RA cohort. In our Risk-RA cohort the finding of increased levels of IL-6 in the Risk-RA phase, something also seen in Pre-RA studies [24, 32], is a sign of inflammation in individuals progressing to arthritis, although the levels are comparable to the levels in healthy controls (Supplementary Fig. S3, available at *Rheumatology* online). The decreased levels of IL-15Rα in individuals progressing to arthritis are, in contrast, significantly different from levels in healthy controls. Decreased levels of IL-15Rα can reflect an increased heterodimeric form of IL-15Rα bound to IL-15, with lower level of the detected free form. IL-15 signalling via trimeric (including IL-15Rα) or dimeric receptor is considered important for activating fibroblast-like synoviocytes as well as immune cells, and promoting chronic inflammation [33, 34]. Notably, targeting IL-15 possibly has an effect on disease activity in RA patients [35]. Our observations of IL-6 and IL-15 raise the question of whether treatment with anti-IL-6 or anti-IL-15 could be effective in a transition from musculoskeletal complaints to chronic joint inflammation.

Finally, ultrasound-detected tenosynovitis turned up as a very strong predictor of arthritis development (median of 11 months later) in the rather few individuals lacking subclinical arthritis at baseline. Previous studies have suggested that tenosynovitis could be part of a subclinical phase of arthritis (including subclinical synovitis) prior to arthritis onset and/or established RA [36–39]. Our finding suggests additional processes in the Risk-RA phase of tendon synovia, affected in the absence of joint synovia involvement. This observation gives support to the notion that ACPA with certain reactivities may be causally involved in the generation of tenosynovitis, something that has been proposed from studies in mice where tenosynovitis in the absence of arthritis was observed after passive transfer of selected monoclonal ACPA [40]. The association of ACPA reactivity and arthritis progression contrasts with a recent study on mice suggesting not just lack of pro-inflammatory effect by ACPA in mice [41] but an anti-inflammatory effect of ACPA in mice with arthritis [13]. These discrepancies could occur for several reasons and further studies of more complex mouse models that better mimic different human risk phases with presence of, for example, ACPA reactivities, other autoantibodies and/or HLA-SE are needed.

In this study, we investigate the biological processes at baseline by statistically taking into count the variable follow-up time and looking for factors with strong and independent contribution to the development of arthritis. This creates simplified pictures of complex biological processes (Fig. 4) and the proposed factors need to be investigated in laboratory studies to be able to dissect the actual role of each factor. We thus identified a prediction model with AUC of 0.82 (95% CI: 0.76, 0.89) that could be cross validated with a lower but overlapping AUC of 0.70 (95% CI: 0.56, 0.85); however, the model needs to be validated in a larger cohort. The identified factors, IL-6 and IL-15Rα, and the relative difference of levels are less suitable for use in a clinical setting to predict disease as they are not available for measurement in clinical labs. Our data also suggest that predictive information will be lost when dichotomizing these factors. On the other hand, we observed that testing for ACPA reactivities can identify one out of four at-risk individuals, with less need of specialist care for follow-up assessment, and ultrasound assessment of tenosynovitis can identify a group of individuals where 9 out of 10 will progress to arthritis suggesting potential for these factors to be measured in the clinic, after further research.

Other limitations of the study are the absence of analyses of bone erosions [42] and the lack of cell phenotyping [43]. These limitations may be mitigated by additional studies of individuals at risk for RA.

## Supplementary Material

keae146_Supplementary_Data

## Data Availability

The data underlying this article cannot be shared publicly due to ethical and legal reasons*.* Condensed data can possible be shared on reasonable request to the corresponding author.
