# Supplementary figures and images for: Identification of early risk factors for anti-citrullinated-protein-antibody positive rheumatoid arthritis—a prospective cohort study
Source: Rheumatology (Oxford). 2024 Mar 8;63(11):3164–71. doi: 10.1093/rheumatology/keae146 (PMC11534094; doi:10.1093/rheumatology/keae146)

**Supplementary Figure S1.**

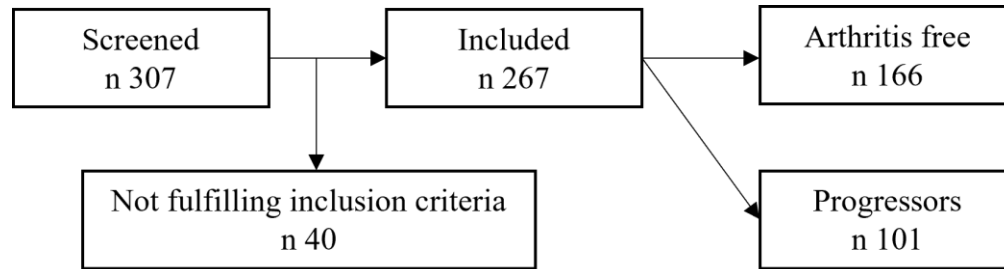

Supplementary Figure S2.

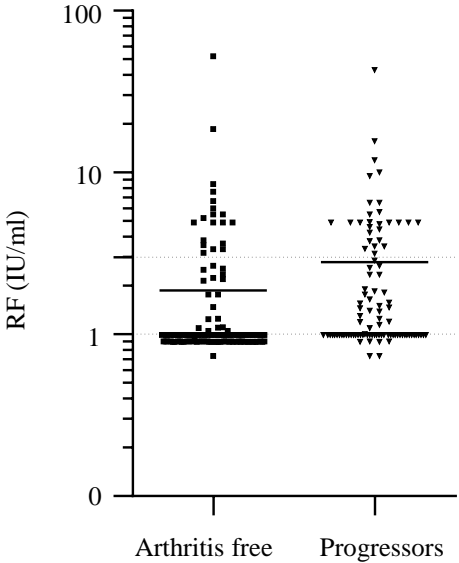

Supplementary Figure S3.

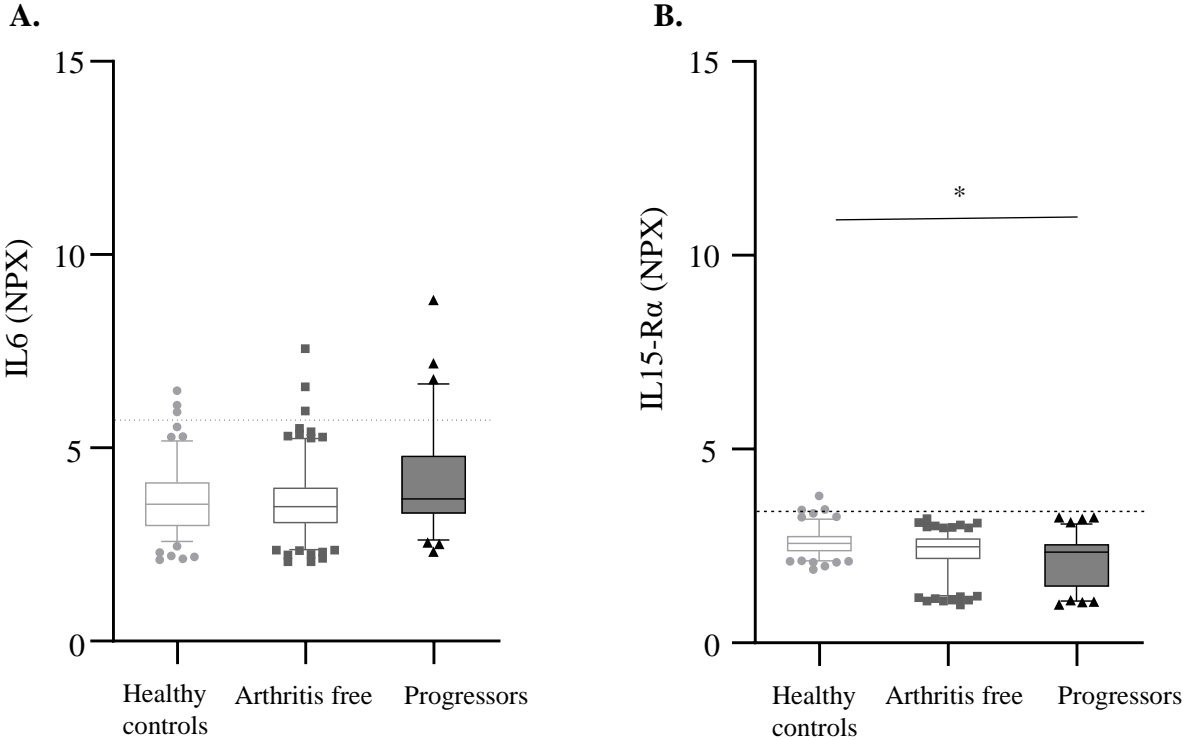

Supplement: keae146_Supplementary_Data [file keae146_supplementary_data.pdf]
